# Prognostic nomogram based on immune scores for breast cancer patients

**DOI:** 10.1002/cam4.2428

**Published:** 2019-07-24

**Authors:** Ju Wang, Yanling Li, Wenying Fu, Ye Zhang, Jun Jiang, Yi Zhang, Xiaowei Qi

**Affiliations:** ^1^ Department of Epidemiology and Biostatistics, West China School of Public Health Sichuan University Chengdu Sichuan P.R. China; ^2^ Department of Breast and Thyroid Surgery, Southwest Hospital Third Military Medical University (Army Medical University) Chongqing P.R. China

**Keywords:** breast cancer, immune scores, nomogram, prognosis

## Abstract

**Background:**

Increased attention has been focused on cancer immunity gene signature. However, the threshold of immune scores to predict disease‐free survival (DFS) and overall survival (OS) in breast cancer has not yet been defined. This study aimed to explore the association of immune scores with prognosis and build a clinical nomogram to predict the survival of patients with breast cancer.

**Methods:**

A total of 986 subjects were analyzed, and clinicopathological characteristics and immune scores were obtained from the TCGA database. Cox proportional hazards regression model was used to estimate the adjusted hazard ratios (*HR*s). Based on results of multivariate analysis, nomograms were built. The models were subjected to bootstrap internal validation. The predictive accuracy and discriminative ability were measured by concordance index (C‐index) and the calibration curve.

**Results:**

The patients were divided into three subgroups according to their immune scores. We found that compared with patients with low immune scores, those with intermediate and high immune scores had significantly improved DFS (*HR* and 95% confidence interval [*CI*]: 0.439 [0.242‐0.799], 0.541 [0.343‐0.855], respectively), whereas only intermediate immune scores significantly indicated better OS (*HR* and 95% *CI*: 0.385 [0.163‐0.910]). The C‐index for DFS and OS prediction was 0.723 (95% *CI*, 0.661‐0.785) and 0.800 (95% *CI*, 0.724‐0.877), respectively. The calibration curves for probability of 3‐ and 5‐year DFS showed significant agreement between nomogram predictions and the actual observations.

**Conclusions:**

High and/or intermediate immune scores are significantly correlated with better DFS and OS in patients with breast cancer. Moreover, the nomograms for predicting prognosis may help to estimate the survival of patients.

## 1 INTRODUCTION

Breast cancer is the most common cancer in women. Over 626 000 deaths and 2 million newly diagnosed cases occur annually worldwide (http://gco.iarc.fr/). It was reported that the estimates of new breast cancer cases were about 278 900 and the estimates of breast cancer deaths were 66 000 in China in 2014 for female.^1^ Though early breast cancer is quite treatable, patients with advanced breast cancer have far greater negative outcomes.^2^ Because of comprehensive treatment including immunotherapy, the prognosis of cancer patients has been greatly improved.^3^, ^4^, ^5^ Therefore, understanding the relationship between the immune system and prognosis is vital to effectively utilize promising immune‐oncology agents.^6^

Recently, increasing attention has been focused on the association between tumor microenvironment and the prognosis of cancer, such as breast cancer^7^ and gastric adenocarcinoma.^8^ It is noticeable that infiltrating immune cells have been associated with tumor growth, invasion and metastasis in some cancers.^9^, ^10^ In accordance with these findings, Ali et al^11^ revealed that immune infiltration is associated with clinical prognosis of breast cancer patients. Furthermore, immune scores which could be calculated from gene expression data were used to indicate immune signatures, even estimate the infiltration of immune cells in tumor tissue.^12^ However, none of these has been sufficiently informative for guidance in clinical practice. Notably, the effectiveness of adopting targeted therapy depending on immune scores, still remains a major clinical issue,^13^ although gene expression profiling has significantly improved the level of comprehensive and individual treatment of breast cancer patients.^14^, ^15^

To the best of our knowledge, there are limited studies that focus on the relationship between immune scores and breast cancer prognosis. We sought to evaluate the association of immune scores with prognosis and built a clinical nomogram for predicting survival of patients with breast cancer.

## 2 MATERIAL AND METHODS

### 2.1 Materials

This study made use of data in the public domain. Data were downloaded from The Cancer Genome Atlas (TCGA) dataset. The details of the TCGA data were described previously^16^ and only a brief introduction is given here. TCGA is currently the largest available dataset for genomic analysis of tumors, including at least 200 kinds of cancer and clinical information, as well as measurements such as DNA methylation, RNA sequencing (https://cancergenome.nih.gov/).

TCGA's clinical pathological information was downloaded from an open‐access resource,^17^ which included the unique number of the patients, age, tumor node metastases (TNM) stage, estrogen receptor (ER), progesterone receptor (PR), DFS time, DFS, OS time and OS etc. Detailed information is available on the following website: http://www.cbioportal.org/.

Immune scores were calculated as previously described.^12^ Briefly, an algorithm was present to calculate immune scores by gene expression data and immune scores were used to estimate the level of infiltrating immune cells. After gene expression profiles of normal hematopoietic samples were compared with these of other normal cells, the overlap that constituted the immune signature was obtained, which represented the infiltration of immune cells in tumor tissue.^12^

### 2.2 Data preprocessing

**Figure 1:**
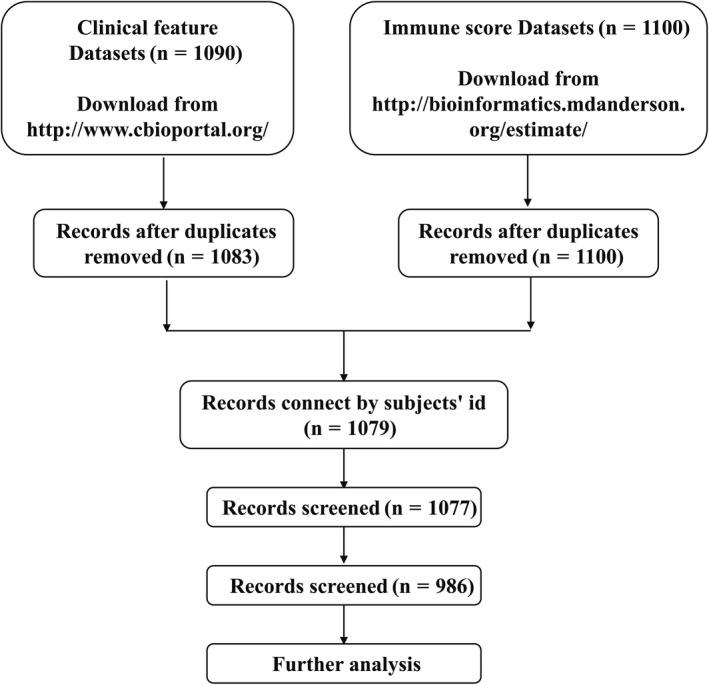
Study flowchart detailing the flow of samples at each stage of analysis

Where replicate cases were identified, all records were removed from further analyses. In total, 1079 cases were available for analysis following the removal of replicate records. Details of sample sizes included at each stage of analysis are listed in Figure 1 as a flowchart. Each immune score corresponds to one patient.

### 2.3 Statistical analysis

Primary end points were OS and DFS. The OS was defined as death from any cause, and DFS was defined as the time prior to relapse of the primary tumor. The cut‐point for immune scores was obtained using X‐tile 3.6.1 software (Yale University School of Medicine, New Haven, CT, USA), as described previously.^18^ X‐tile plots were conducted for assessment of immune scores; this was expressed as optimization of cut points based on outcome.^18^ Categorical data were analyzed using *Chi*‐square test or Fisher's exact test, and continuous variables were analyzed using the analysis of variance test (ANOVA) or the Kruskal‐Wallis H test for variables with an abnormal distribution and homogeneity of variance. Survival curves were constructed using the Kaplan‐Meier method and were compared using the log‐rank test. This was done to explore the differences between immune scores subgroups and prognosis (DFS and OS) using the GraphPad Prism 6.0 software (GraphPad Software Inc, La Jolla, CA, USA). Multivariate Cox proportional hazards regression model was used to identify the independent predictors of DFS and OS. After the effect of age, ER, PR, HER2 and TNM stage were simultaneously considered, adjusted Hazard ratios and 95% confidence interval (*CI*) were estimated.

Nomograms were formulated based on the results of multivariate analysis using R version 3.5.1 (http://www.r-project.org). These nomograms were subjected to 1000 bootstrap resamples for internal validation of the analyzed database. The performance of models for predicting prognosis was evaluated by calculating the concordance index (C‐index).^19^ The value of the C‐index was between 0.5 and 1.0, with 1.0 indicating the perfect ability to correctly discriminate the outcomes with the model and 0.5 indicating a random chance. Calibration of the nomogram for 3‐, and 5‐year DFS was performed by comparing the observed survival with the predicted survival probability.

All statistical tests were two‐sided and *P* values of <.05 were considered statistically significant. Data compilations and descriptive statistics were performed using the SAS 9.3 software (SAS Institute Inc, Cary, NC, USA).

## 3 RESULTS

### 3.1 Patients’ characteristics

A total of 986 patients were included in our analysis datasets after data cleaning (for specific data preparation, see Figure 1). The average age of patients was 57.84 years (SD = 12.92, range 26‐90), and 679 (68.86%) patients were older than 50 years. Of the 986 patients, 768 (77.89%) patients were ER positive, 674 (68.36%) were PR positive, and 570 (57.81%) were TNM stage II. Median immune scores of patients were 119.86 (range −1559.28‐3459.35, interquartile range 1075). The cut points of immune scores were −534.7 and −100.1, thus patients were subsequently subdivided into high, intermediate and low immune scores subgroups (X‐tile plots are shown in the Figure S1). Totally, 176 (17.85%) patients were lower than or equal to −534.7 (low immune scores subgroup), 203 (20.59%) were between −534.7 and −100.1 (intermediate immune scores subgroup), and 607 (61.56%) patients were greater than −100.1 (high immune scores subgroup). The median DFS time was 24.64 months (range 0‐281.08 months) and the median OS time was 25.76 months (range 0‐282.69 months).

Table 1 presents the clinicopathologic characteristics of the different subgroups according to immune scores. The average ages of different immune scores subgroups were 59 (SD = 13.49), 57 (SD = 12.41) and 57 (SD = 12.93), respectively. As for ER, PR and HER2 status, the proportion of those that were negative was higher in the high immune scores subgroup compared with the low scores subgroup. Compared with low immune scores subgroup, the patients with intermediate and high immune scores tended to be staged in II and III.

**Table 1:** Associations between clinical pathological characteristics and immune scores in 986 breast cancer patients

Characteristics	Total	Immune scores			*χ* ^2^ value	*P* value
		≤−534.7	−534.7 to −100.1	>−100.1		
Sample sizes	986	176 (17.85)	203 (20.59)	607 (61.56)	—	—
Age (y)^a^					13.043	0.221
≤40	91	18 (10.23)	21 (10.34)	52 (8.57)		
40‐50	216	28 (15.91)	37 (18.23)	151 (24.88)		
50‐60	251	45 (25.57)	57 (28.08)	149 (24.55)		
60‐70	257	49 (27.84)	55 (27.09)	153 (25.21)		
70‐80	129	26 (14.77)	29 (14.29)	74 (12.19)		
>80	42	10 (5.68)	4 (1.97)	28 (4.61)		
ER					9.853	0.007
Negative	218	31 (17.61)	33 (16.26)	154 (25.37)		
Positive	768	145 (82.39)	170 (83.74)	453 (74.63)		
PR					5.039	0.081
Negative	312	58 (32.95)	51 (25.12)	203 (33.44)		
Positive	674	118 (67.05)	152 (74.88)	404 (66.56)		
HER2					3.802	0.149
Negative	703	121 (68.75)	136 (67.00)	446 (73.48)		
Positive	283	55 (31.25)	67 (33.00)	161 (26.52)		
TNM stage					7.204	0.302
I	167	35 (19.89)	33 (16.26)	99 (16.31)		
II	570	100 (56.82)	121 (59.61)	349 (57.50)		
III	227	35 (19.88)	42 (20.69)	150 (24.71)		
IV	22	6 (3.41)	7 (3.44)	9 (1.68)		

Abbreviations: ER, estrogen receptor; HER2, human epidermal growth factor receptor 2; PR, progesterone receptor.

a Age at diagnosis of breast cancer.

### 3.2 Univariate and multivariate analyses for DFS and OS

**Figure 2:**
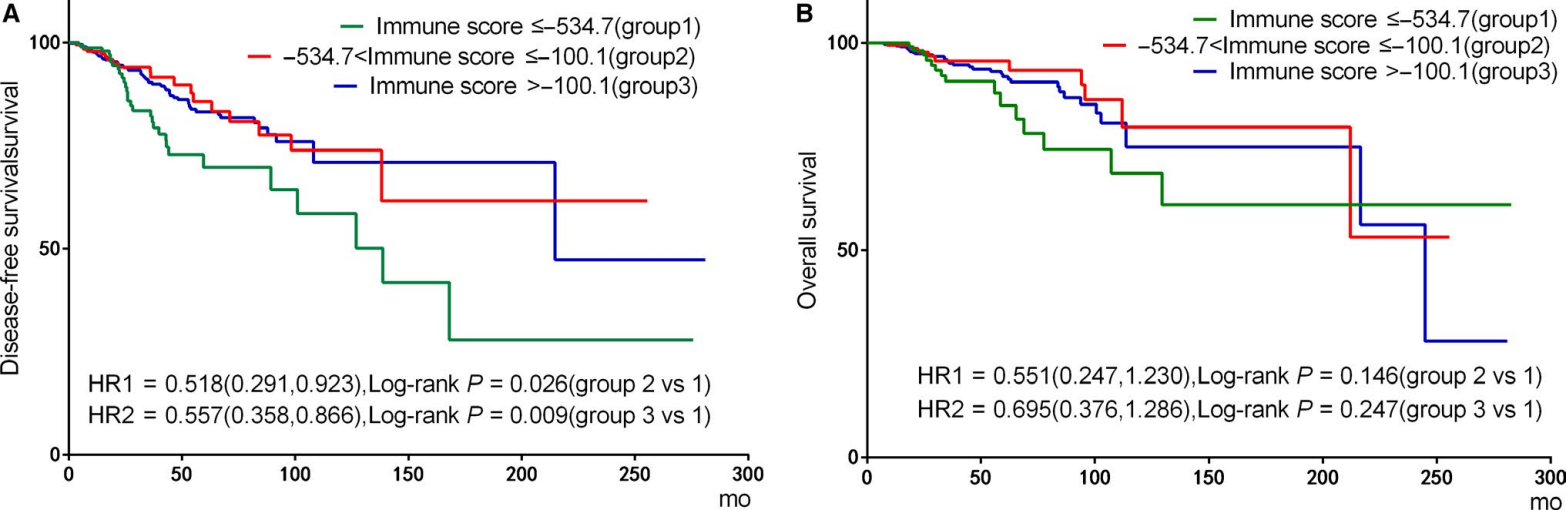
Kaplan‐Meier curves depicting associations of immune scores subgroups with disease‐free survival (DFS) and overall survival (OS) for patients with breast cancer. Comparison of DFS (A) and OS (B) among patients with ≤−534.7 immune scores (group 1), patients with immune scores between −534.7 and −100.1 (group 2), and patients with>−100.1 immune scores (group 3)

Table 2 displays the unadjusted associations between clinical pathological characteristics and prognosis. As shown in Table 2 and Figure 2, there were significant differences in DFS among patients with low, intermediate and high immune scores (hazard ratios [*HR*]: 0.518, 95% confidence interval [*CI*], 0.291‐0.923, *P* = .026; *HR*: 0.557, 95% *CI*, 0.358‐0.866, *P* = .009, respectively), while no significant differences were found for OS (*HR*: 0.551, 95% *CI*, 0.247‐1.230, *P* = .146; *HR*: 0.695, 95% *CI*: 0.376‐1.286, *P* = .247, respectively). In addition, ER positive, PR positive, HER2 negative and low TNM stage were statistically associated with longer DFS and OS, respectively (*P* < .05).

**Table 2:** Univariate analyses of OS and DFS among breast cancer patients according to clinic pathological characteristics and immune scores

Characteristics	Total	DFS				OS			
		Nonrelapse	Relapse	*HR* (95% CI)	*P* value	Survival	Deadth	*HR* (95% CI)	*P* value
Age (y)^a^									
≤40	91	72 (8.22)	19 (17.27)	1.000		81 (8.75)	10 (16.67)	1.000	
40‐50	216	191 (21.80)	25 (22.73)	0.494 (0.271, 0.901)	0.021	201 (21.71)	15 (25.00)	0.605 (0.271, 1.351)	0.220
50‐60	251	231 (26.37)	20 (18.18)	0.424 (0.226, 0.796)	0.008	240 (25.92)	11 (18.33)	0.508 (0.215, 1.198)	0.122
60‐70	257	229 (26.14)	28 (25.45)	0.638 (0.356, 1.146)	0.133	243 (26.24)	14 (23.33)	0.734 (0.325, 1.659)	0.457
70‐80	129	117 (13.36)	12 (10.91)	0.623 (0.302, 1.286)	0.201	122 (13.17)	7 (11.67)	0.902 (0.340, 2.390)	0.835
>80	42	36 (4.11)	6 (5.45)	1.644 (0.649, 4.169)	0.295	39 (4.21)	3 (5.00)	2.370 (0.637, 8.815)	0.198
Immune scores									
≤−534.7	176	146 (16.67)	30 (27.27)	1.000		161 (17.39)	15 (25.00)	1.000	
−534.7 to −100.1	203	184 (21.00)	19 (17.28)	0.518 (0.291, 0.923)	0.026	193 (20.84)	10 (16.67)	0.551 (0.247, 1.230)	0.146
>−100.1	607	546 (62.33)	61 (55.45)	0.557 (0.358, 0.866)	0.009	572 (61.77)	35 (58.33)	0.695 (0.376, 1.286)	0.247
ER									
Negative	218	183 (20.89)	35 (31.82)	1.000		194 (20.95)	24 (40.00)	1.000	
Positive	768	693 (79.11)	75 (68.18)	0.622 (0.416, 0.929)	0.020	732 (79.05)	36 (60.00)	0.472 (0.28, 0.796)	0.005
PR									
Negative	312	263 (30.02)	49 (44.55)	1.000		282 (30.45)	30 (50.00)	1.000	
Positive	674	613 (69.98)	61 (55.45)	0.585 (0.401, 0.852)	0.005	644 (69.55)	30 (50.00)	0.513 (0.308, 0.853)	0.010
HER2									
Negative	703	635 (72.49)	68 (61.82)	1.000		668 (72.14)	35 (58.33)	1.000	
Positive	283	241 (27.51)	42 (38.18)	1.327 (0.892, 1.974)	0.163	258 (27.86)	25 (41.67)	1.287 (0.747, 2.216)	0.363
TNM stage									
I	167	157 (17.92)	10 (9.09)	1.000		164 (17.71)	3 (5.00)	1.000	
II	570	521 (59.47)	49 (44.55)	1.869 (0.946, 3.692)	0.072	546 (58.96)	24 (40.00)	2.995 (0.901, 9.949)	0.073
III	227	187 (21.35)	40 (36.36)	4.264 (2.131, 8.535)	<0.001	203 (21.92)	24 (40.00)	8.074 (2.428, 26.849)	0.001
IV	22	11 (1.26)	11 (10.00)	11.624 (4.882, 27.673)	<0.001	13 (1.41)	9 (15.00)	22.13 (5.909, 82.887)	<0.001

Abbreviations: CI, confidence interval; ER, estrogen receptor; HER2, human epidermal growth factor receptor 2; HR, hazard ratio; PR, progesterone receptor.

a Age at diagnosis of breast cancer.

Results of the multivariate Cox proportional hazard regression analyses are shown in Table 3. Compared with patients with low immune scores, those with intermediate and high immune scores had significantly improved DFS (*HR* and 95% confidence interval (*CI*): 0.439 [0.242‐0.799] and 0.541 [0.343‐0.855], respectively). Meanwhile, only the intermediate immune scores subgroup was significantly correlated with improved OS (*HR*: 0.385, 95% *CI*: 0.163‐0.910). As expected, when compared with patients in stage I, patients in stages III and IV had significantly poorer DFS (*HR* and 95% *CI* in stages II, III and IV were 2.023 [1.017‐4.022], 4.749 [2.344‐9.620] and 11.566 [4.539‐29.474], respectively), and similar results were obtained for OS (*HR* and 95% *CI* in stages II, III and IV were 3.519 [1.040‐11.907], 10.346 [3.038‐35.231] and 28.687 [6.909‐119.124], respectively). Interestingly, when compared with patients who were younger than 40 years of age, patients who were 50‐60 years of age conferred better DFS (*HR*: 0.497, 95% *CI*: 0.263‐0.942). As for the rest of the clinical characteristics, significant associations were not recognized.

**Table 3:** Multivariate analyses of OS and DFS among breast cancer patients according to clinical characteristics and immune scores

Characteristics	DFS		OS	
	*HR* (95%*CI*)	*P* value	*HR* (95%*CI*)	*P* value
Age (y)^a^				
≤40	1.000		1.000	
40‐50	0.605 (0.325, 1.129)	0.114	0.703 (0.301, 1.643)	0.416
50‐60	0.497 (0.263, 0.942)	0.032	0.637 (0.264, 1.538)	0.316
60‐70	0.703 (0.374, 1.322)	0.274	0.697 (0.277, 1.759)	0.445
70‐80	0.649 (0.310, 1.361)	0.253	0.961 (0.353, 2.617)	0.939
>80	1.737 (0.648, 4.651)	0.272	2.413 (0.555, 10.493)	0.240
Immune scores				
≤−534.7	1.000		1.000	
−534.7 to −100.1	0.439 (0.242, 0.799)	0.007	0.385 (0.163, 0.910)	0.030
>−100.1	0.541 (0.343, 0.855)	0.008	0.671 (0.349, 1.288)	0.230
ER				
Positive	1.000		1.000	
Negative	1.192 (0.657, 2.163)	0.562	2.096 (0.880, 4.989)	0.095
PR				
Positive	1.000		1.000	
Negative	1.533 (0.882, 2.666)	0.130	1.209 (0.521, 2.804)	0.659
HER2				
Positive	1.000		1.000	
Negative	1.110 (0.725, 1.699)	0.630	1.234 (0.683, 2.229)	0.485
TNM stage				
I	1.000		1.000	
II	2.023 (1.017, 4.022)	0.044	3.519 (1.040, 11.907)	0.043
III	4.749 (2.344, 9.620)	<0.001	10.346 (3.038, 35.231)	<0.001
IV	11.566 (4.539, 29.474)	<0.001	28.687 (6.909, 119.124)	<0.001

Abbreviations: CI, confidence interval; ER, estrogen receptor; HER2, human epidermal growth factor receptor 2; HR, hazard ratio; PR, progesterone receptor.

a Age at diagnosis of breast cancer.

### 3.3 Prognostic nomogram for DFS and OS

**Figure 3:**
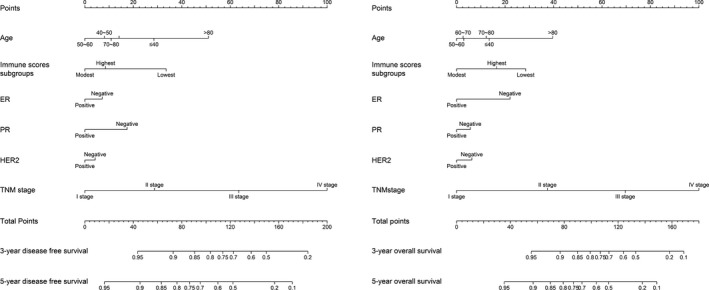
Breast cancer survival nomograms. (For using the nomograms, an individual patient's value is located on each variable axis, and a line is drawn upward to determine the number of points received for each variable value. The sum of these numbers is located on the Total Points axis, and a line is drawn downward to the survival axes to determine the likelihood of 3‐ or 5‐year survival.)

**Figure 4:**
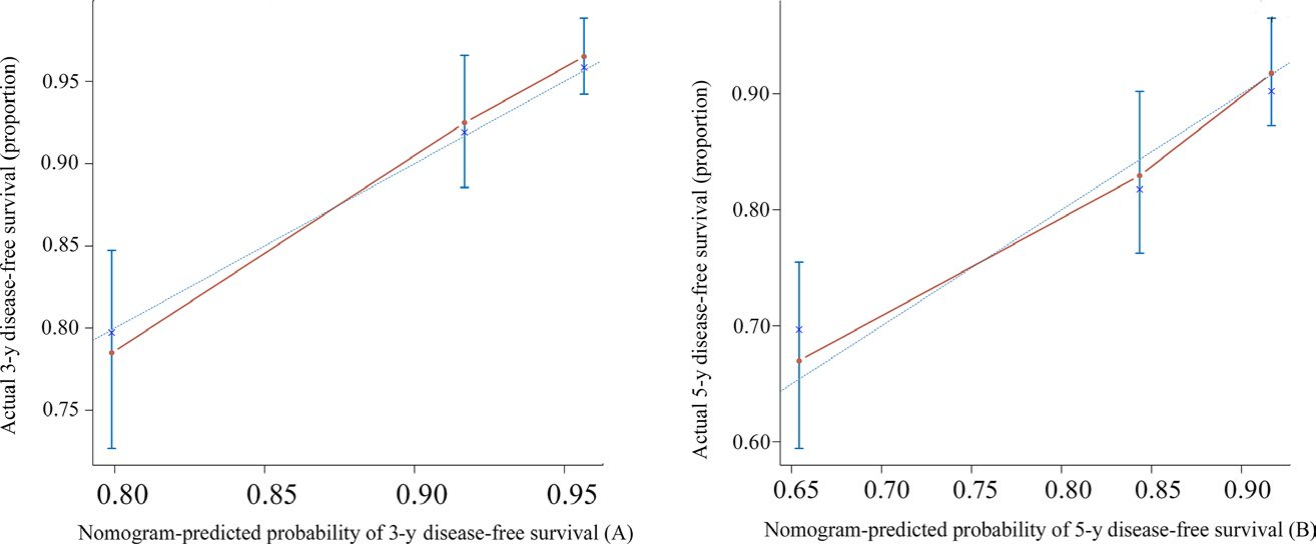
The calibration curve of disease‐free survival (DFS) at 3 and 5 years for the breast cancer. Nomogram‐predicted probability of DFS is plotted on the *x*‐axis; actual DFS is plotted on the *y*‐axis. The calibration curve for predicting overall survival was not shown here

The prognostic nomogram that integrated all considered independent factors for DFS and OS are shown in Figure 3. The C‐index for DFS and OS predictions were 0.723 (95% *CI*, 0.661‐0.785) and 0.800 (95% *CI*, 0.724‐0.877), respectively. The calibration plot for the probability of survival at 3‐ or 5‐ year showed good agreement between the prediction by nomograms and actual observations (Figure 4A and B).

## 4 DISCUSSION

In the present study, we evaluated the prognostic significance of immune scores by using gene expression data in patients with breast cancer. After possible confounders were considered, we found that high and/or intermediate immune scores were significantly associated with DFS and OS of breast cancer patients. Meanwhile, we also built nomograms to easily predict the survival of patients with breast cancer.

The contribution of immune cells to breast cancer has been well recognized,^10^, ^20^ and immunity gene signature is considered as a biomarker for immunotherapy responses.^12^ In addition, previous studies have shown that immunology gene signature significantly correlated with prognosis of breast cancer.^15^, ^21^ A study^15^ found that immunity gene expression should be incorporated into the current multi‐gene assays to improve assessment of prognosis of breast cancer patients. However, they have not yet been applied for the prediction of DFS and OS probability in clinical studies. Moreover, nomograms, which took immune scores into account, were sparse. In our study, based on TCGA datasets, the clinical pathological information and immune scores of breast cancer patients were used to explore the relationship between immune scores and prognosis. Furthermore, nomograms were also built to estimate the prognosis of patients with breast cancer easily.

When adjusted for possible confounders, higher immune scores significantly conferred better DFS and OS in breast cancer patients. The possible reason is that higher immune scores indicated an enhanced immune system and function, which could be mobilized to increase the antitumor immunity of tumor microenvironments, so as to control and eliminate the tumor.^5^, ^22^ Furthermore, important genes, such as CD302, which were used to compute immune signatures, played critical roles in immune function.^12^ In addition, a study^23^ revealed that expression of T cell‐related marker, CD3D, was associated with higher pathologic complete response in patients with breast cancer who received neoadjuvant chemotherapy. Therefore, immune scores may not only be used as prognostic biomarker for breast cancer patients, but also have potential clinical values in the choice of therapeutic strategies.^22^, ^24^ Importantly, we found that patients with higher immune scores tended to be ER, PR, and HER2 negative. It implied that subjects might be less responsive to treatments with antiestrogen and anti‐HER2 therapy, while they may benefit from immunotherapies to further improve survival.^15^, ^25^, ^26^ Generally speaking, patients with ER or PR positive and HER2 negative status had better prognosis than those with ER or PR negative and HER2 positive status, respectively, which was consistent with our results from univariate analyses. However, in multivariate analyses, no statistical associations between ER/PR/HER2 status and prognosis were obtained, and the possible reasons are listed as followings. Firstly, novel and efficacious approaches, including aromatase inhibitor, fulvestrant and CDK4/6 inhibitor ER/PR positive patients, trastuzumab, pertuzumab and trastuzumab emtansine for HER2 positive patients, platinum, PD1/PDL1 inhibitor and PARP inhibitor for ER/PR/HER2 negative patients, have greatly enriched the comprehensive treatment of breast cancer, and improved the prognosis of breast cancer patients.^27^, ^28^ The intrinsic characteristics of breast cancer are being changed by the above therapeutic approaches. For example, in the latest pathological prognostic stage system, HER2 positive status was regarded as an indicator for good prognosis.^29^ In addition, patients who were 50‐60 years of age conferred better DFS (*HR*: 0.497, 95% *CI*: 0.263‐0.942), when compared with patients younger than or equal to 40 years. A possible explanation for the result is that young females may have larger tumors, higher‑grade tumors, lymph node positivity and a tendency towards reduced DFS.^30^

To the best of our knowledge, these are the first nomograms for predicting OS and DFS of patients with breast cancer that are based on immune scores and clinicopathologic characteristics. Through these ready‐to‐use scoring systems, both patients and physicians can achieve an individualized survival prediction. Identifying subgroups of individuals at different risks for poor survival might have an effect on treatment option. However, datasets including gene expression files that could be used to calculate immune scores are sparse. Therefore, our nomograms were limited by the validation of external data. Further efforts to collect data relating to immune gene expression, in addition to incorporating clinicopathological factors are encouraged to further develop our models. Furthermore, limited by lack of the treatment information of breast cancer in the TCGA dataset, we were unable to adjust for the effect of treatment on prognosis. Further study are encourged to collect these personal characteristics to improve and verify our models.

## 5 CONCLUSION

Our findings indicate that high and/or intermediate immune scores are significantly correlated with better DFS and OS in patients with breast cancer. Also, we established and validated novel nomograms for predicting prognosis. This practical prognostic model may help easily estimate the survival of patients, as well as identify subgroups of patients who are in need of aggressive adjuvant therapy.

## CONFLICT OF INTEREST

None declared.
