# Supplementary figures and images for: Prognostic nomogram based on immune scores for breast cancer patients
Source: Cancer Med. 2019 Jul 24;8(11):5214–22. doi: 10.1002/cam4.2428 (PMC6718583; doi:10.1002/cam4.2428)

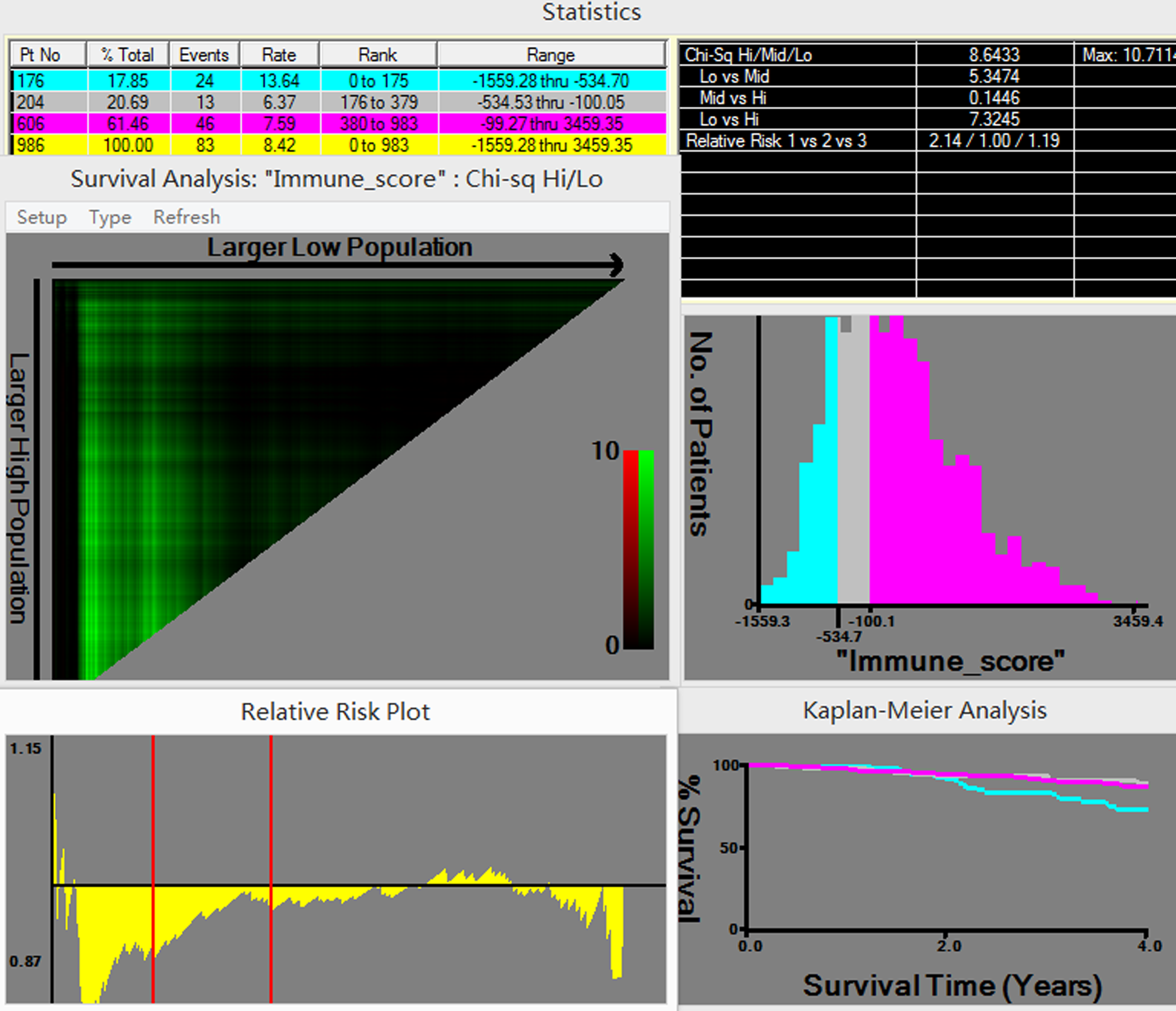

Supplement: Supplementary file 1 [file CAM4-8-5214-s001.tif]
